# Supplementary material for: The Pictorial Screening Memory Test (P-MIS) for Adults with Moderate Intellectual Disability and Alzheimer’s Disease
Source: Int J Environ Res Public Health. 2022 Aug 30;19(17):10780. doi: 10.3390/ijerph191710780 (PMC9518372; doi:10.3390/ijerph191710780)
Supplement: Supplementary file 1 [file ijerph-19-10780-s001.zip › ijerph-1839223-supplementary.pdf]

Comparisons of the area under the curve of PMIS-ID total scores for MCI and AD in people with moderate level of ID.

Table S1. Paired comparisons of the areas under the curve of the PMIS-ID trials diagnostic accuracy.

Paired results	z	Bilateral signification	AUC difference	SE difference	CI 95 % (asymptotic) Lower limit	Upper limit
MCI						
TIR - TPMISID	0.38	0.970	0.04	0.474	-0.181	0.188
AD						
TIR – TDR	-0.842	0.400	-0.073	0.398	-0.242	0.096
TIR – TPMISID	-1.021	0.307	-0.064	0.398	-0.188	0.059
TDR -TPMISID	0. 229	0.819	-0.008	0.376	-0. 063	0.080

MCI, Mild Cognitive Impairment; TIR, Total immediate recall, TPMISID, Total picture memory impairment screen for people with intellectual disability; AD: Alzheimer's disease; TDR, Total delayed recall; AUC, Area under the curve; SE, Standard error; CI, confidence interval,

Normative data of the PMIS-ID delayed and total recall for people with AD and moderate level of ID.

Table S2. Normative data of the PMIS-ID delayed total score for different cut-off points for AD in moderate level of ID sample.

Cut-off points	S	Sp	*J*	PPV[a]	NPV[a]
0	0.00	1.00	0.00	0.64	1.00
0.5	0.12	0.96	0.08	0.70	0.94
1.5	0.18	0.93	0.11	0.71	0.89
2	0.31	0.86	0.17	0.71	0.80
2.5	0.37	0.86	0.23	0.76	0.80
3	0.37	0.79	0.16	0.74	0.73
4.5	0.50	0.72	0.22	0.78	0.67
5	0.50	0.69	0.19	0.77	0.64
6	0.62	0.65	0.27	0.79	0.62
6.5	0.66	0.62	0.28	0.86	0.59
7.5	0.75	0.37	0.12	0.85	0.47
8	1.00	0.00	0.00	1.00	0.36

PMIS-ID, Picture Memory Impairment Screen for people with Intellectual Disability; S, Sensitivity; Sp, Specificity; *J*, Youden's J statistic; PPV, Positive predictive values; NPV, Negative predictive values.

[a]25.5 % is the prevalence of AD in the sample.

Table S3. Normative data of the PMIS-ID total score for different cut-off points for AD in moderate level of ID sample.

Cut-off points	S	Sp	*J*	PPV[a]	NPV[a]
0	0.00	1.00	0.00	0.64	1.00
0.5	0.12	1.00	0.12	0.66	1.00
1	0.12	1.00	0.12	0.69	1.00
2	0.19	1.00	0.19	0.71	1.00
3	0.19	1.00	0.19	0.72	1.00
4	0.25	0.96	0.21	0.72	0.94
5.5	0.31	0.96	0.27	0.74	0.94
7	0.31	0.93	0.24	0.73	0.89
8.5	0.37	0.90	0.27	0.72	0.84
9	0.37	0.86	0.23	0.74	0.80
9.5	0.56	0.83	0.39	0.73	0.76
10	0.56	0.79	0.35	0.72	0.73
10.5	0.56	0.79	0.35	0.79	0.73
11	0.62	0.69	0.31	0.77	0.64
11.5	0.62	0.65	0.27	0.79	0.62
12.5	0.66	0.62	0.28	0.78	0.59
13	0.69	0.59	0.28	0.81	0.57
13.5	0.69	0.59	0.28	0.85	0.57
14	0.75	0.48	0.23	0.82	0.52
14.5	0.81	0.44	0.25	0.87	0.50
15.5	0.94	0.27	0.21	0.80	0.43
16	1.00	0.00	0.00	1.00	0.36

PMIS-ID, Picture Memory Impairment Screen for people with Intellectual Disability; S, Sensitivity; Sp, Specificity; *J*, Youden's J statistic; PPV, Positive predictive values; NPV, Negative predictive values.
[a]25.5 % is the prevalence of AD in the sample.

Normative data of the PMIS-ID immediate, delayed and total recall for people with AD and mild level of ID.

Table S4. Normative data of the PMIS-ID immediate total score for different cut-off points for AD in mild level of ID sample.

Cut-off points	S	Sp	*J*	PPV[a]	NPV[a]
<2	0.00	1.00	0.00	0.00	0.14
3	0.70	0.80	0.50	0.92	0.25
3.5	0.84	0.60	0.44	0.93	0.38
4.5	0.87	0.60	0.47	0.93	0.43
5	0.90	0.60	0.50	0.90	0.40
5.5	0.94	0.60	0.54	0.91	0.50
6	0.97	0.40	0.37	0.91	0.67
6.5	1.00	0.40	0.40	0.91	1.00
7.5	1.00	0.40	0.40	0.89	1.00
8	1.00	0.00	0.00	0.86	1.00

PMIS-ID, Picture Memory Impairment Screen for people with Intellectual Disability; S, Sensitivity; Sp, Specificity; *J*, Youden's J statistic; PPV, Positive predictive values; NPV, Negative predictive values.
[a]25.5 % is the prevalence of AD in the sample.

Table S5. Normative data of the PMIS-ID delayed total score for different cut-off points for AD in mild level of ID sample.

**Cut-off points**	**S**	**Sp**	***J***	**PPV[a]**	**NPV[a]**
0	0.00	1.00	0.00	0.00	0.14
0.5	0.62	1.00	0.62	0.86	0.20
2	0.84	0.80	0.84	0.90	0.38
3.5	0.90	0.60	0.50	0.90	0.50
4.5	0.90	0.60	0.50	0.93	0.40
5.5	0.94	0.40	0.50	0.94	0.50
6.5	0.94	0.40	0.34	0.97	0.50
7.5	0.94	0.40	0.34	1.00	0.50
8	1.00	0.00	0.00	1.00	1.00

PMIS-ID, Picture Memory Impairment Screen for people with Intellectual Disability; S, Sensitivity; Sp, Specificity; *J*, Youden's J statistic; PPV, Positive predictive values; NPV, Negative predictive values.
[a]25.5 % is the prevalence of AD in the sample.

Table S6. Normative data of the PMIS-ID total score for different cut-off points for AD in mild level of ID sample.

Cut-off points	S	Sp	*J*	PPV[a]	NPV[a]
<4	0.00	1.00	0.00	0.00	0.14
5	0.55	0.80	0.35	0.89	0.18
7	0.68	0.60	0.84	0.91	0.23
9.5	0.80	0.60	0.40	0.93	0.33
11	0.84	0.40	0.24	0.93	0.38
12	0.88	0.40	0.28	0.93	0.43
12.5	0.90	0.40	0.30	0.93	0.50
13.5	0.90	0.40	0.30	0.90	0.40
14.5	1.00	0.40	0.40	0.91	1.00
15.5	1.00	0.40	0.40	0.89	1.00
16	1.00	0.00	0.00	0.86	1.00

PMIS-ID, Picture Memory Impairment Screen for people with Intellectual Disability; S, Sensitivity; Sp, Specificity; *J*, Youden's J statistic; PPV, Positive predictive values; NPV, Negative predictive values.

[a]25.5 % is the prevalence of AD in the sample.
